# Hypertension Treatment, Blood Pressure, and Deprescribing Among US Nursing Home Residents With and Without Dementia Before and After the COVID-19 Pandemic

**DOI:** 10.1016/j.jamda.2025.105883

**Published:** 2025-10-10

**Authors:** Sirui Zhang, Andrew R. Zullo, Vincent Mor, Sarah D. Berry, Lexie R. Grove, Arman Oganisian, Kaleen N. Hayes

**Affiliations:** a Department of Epidemiology, Brown University School of Public Health, Providence, RI, USA; b Department of Health Services, Policy, and Practice, Brown University School of Public Health, Providence, RI, USA; c Department of Medicine, Beth Israel Deaconess Medical Center, Boston, MA, USA; d Hinda and Arthur Marcus Institute for Aging Research, Hebrew SeniorLife, Boston, MA, USA; e Department of Biostatistics, Brown University School of Public Health, Providence, RI, USA

**Keywords:** Nursing homes, dementia, hypertension, polypharmacy

## Abstract

**Objectives:**

To evaluate hypertension (HTN) management and antihypertensive deprescribing among US nursing home (NH) residents with and without dementia, before and after the onset of the COVID-19 pandemic.

**Design:**

Retrospective cohort study.

**Setting and Participants:**

Newly admitted NH residents with HTN treated with an antihypertensive medication who were not receiving hospice from >1100 NHs using a common electronic health record vendor (January 2, 2018-July 3, 2022) (N = 141,630).

**Methods:**

During an 8-week follow-up period beginning on day 14 in the NH after admission (index date), we measured average blood pressure (BP), BP variability, and prevalence of hypo-/hypertension stratified by dementia status and pre— and post—COVID-19 onset (admission before vs on/after November 3, 2020). We measured antihypertensive medication deprescribing (dose/frequency decreases or cessation for ≥7 days) during a 90-day follow-up and estimated hazard ratios by dementia status using Cox hazards regression models adjusted for demographics and comorbidities. Residents were followed until their first deprescribing event or censoring (discharge, death, 90 days, or March 7, 2022).

**Results:**

BP monitoring was similar between those with and without dementia, but increased post—COVID-19 (eg, 1.6 readings per day pre—COVID-19 vs 1.9 readings per day post—COVID-19 in residents with dementia). Residents with severe dementia had a higher prevalence of hypotension compared with residents without dementia (eg, 16.3% vs 8.9% with at least 1 systolic BP <90 mm Hg among those treated with 2 classes). Residents with dementia were less likely to experience deprescribing than those without dementia (fully adjusted hazard ratio, 0.83; 95% CI, 0.79-0.86).

**Conclusions and Implications:**

Findings suggest potential overtreatment of HTN among residents with dementia and increased BP monitoring during the COVID-19 pandemic. Results highlight potential opportunities for deprescribing to balance BP control and medication, and monitoring burden in NH.

Over 80% of older adults in nursing homes (NHs) have hypertension (HTN)^1,2^; this prevalence is >90% for residents with Alzheimer’s disease and related dementias (ADRD).^3,4^ The management of HTN in the NH settings is challenging due to the high prevalence of multimorbidity, which increases the risk of both cardiovascular events from high blood pressure (BP) and adverse events from HTN medications. Residents with ADRD are at particularly high risk of adverse events related to HTN treatment because they have more difficulty communicating early symptoms of hypotension. Furthermore, the benefits and harms of antihypertensive treatment in older adults with cognitive impairments and limited life expectancy are not well defined, complicating treatment decisions.^5^ The COVID-19 outbreak has been well documented to have shifted priorities away from chronic disease care in NHs across various studies due to system-level disruptions (eg, staffing shortages, clinic closures), provider-level challenges (eg, limited treatment adjustments, care prioritization), and patient-level barriers (eg, fear of infection, reduced adherence).^6-12^ Understanding how these changes have influenced HTN management and outcomes in the NH is critical for optimizing care in this setting because most existing evidence on treatment and its effects is in healthier, community-dwelling populations.^13,14^

In this retrospective cohort study, we aimed to describe HTN management among US NH residents, with a particular focus on differences between residents with and without dementia and the impacts of the COVID-19 pandemic. Our secondary aim was to estimate whether having dementia was associated with a different practice of deprescribing antihypertensive medications in NH. In our study, we hypothesized that the pandemic would alter BP monitoring patterns and increase BP variability, with stronger effects among residents with dementia due to their heightened vulnerability to care disruptions. We further hypothesized that residents with dementia would be less likely to experience deprescribing.

## Methods

### Data Sources and Collection

This project leveraged data from >1100 NHs (accounting for approximately 11% of all US NHs) that used PointClickCare as their electronic health record (EHR) vendor from January 2018 to July 2022. The NH EHR data include resident demographics, vitals, antihypertensive medication use, clinical testing, and other health care—related data. It provides detailed records on barcoded and time stamped electronic medication administration records (eMAR), regular BP measurements, immunization records, and other clinical information. Additionally, this dataset includes Minimum Dataset (MDS) 3.0 information. The MDS is a federally mandated clinical assessment for residents in Medicare-/Medicaid-certified NHs, capturing a wide range of data on demographics; clinical conditions; functional, psychological, and cognitive status; services; and treatments.^15^

### Study Population

The study period was from January 2, 2018, to July 3, 2022. We first identified residents with at least 1 administration of an antihypertensive medication (angiotensin-converting enzyme inhibitors, angiotensin-receptor blockers, calcium channel blockers, thiazide diuretics, or beta-blockers) (Supplementary Table 1), and who had at least 1 baseline MDS assessment within 14 days of admission. Beta-blockers were included because they are commonly prescribed in NH settings for HTN management.^16^ The study index date (time zero) was day 14 after admission. We then excluded residents without a documented HTN diagnosis on their baseline MDS assessment; residents entering from or receiving hospice care during the first 14 days since admission; residents whose first antihypertensive medication was not administered within 14 days of admission; residents without at least 1 recorded BP measurement within the 14 days of admission; and residents who died or were discharged by the index date.

### BP and Antihypertensive Medications

The EHR’s eMAR and orders data were extracted to measure antihypertensive medication exposures, including time and date of administration, medications used (medications in Supplementary Table 1), drug strength, and administration directions (eg, 50 mg twice daily for the treatment of HTN). Importantly, the eMAR includes records of medications administered during the post-acute period covered under Medicare’s Skilled Nursing Facility benefit (which are not captured in Medicare Part D data). We used EHR vital sign datasets that contain separate measurements for systolic BP (SBP) and diastolic BP (DBP), for the main analysis. Each BP measurement is time stamped to the second. After preliminary assessment for plausible values, we first recoded outliers in the top 1% of BP values by truncating both SBP and DBP at their respective 99th percentiles. There were no implausible values in the lowest 1% of SBP and DBP. Baseline BP measurements used values from days 0 to 14 after NH admission. Given the limited number of documented hypertensive urgencies or emergencies measured in the EHR, we chose to focus on instances of elevated BP more broadly. Based on clinical judgment, we defined uncontrolled HTN as an SBP >160 mm Hg—10 mm Hg higher than the 150 mm Hg guideline threshold—to capture individuals at higher risk of complications. Hypotension was defined as an SBP <90 mmHg.^17^

### Outcomes and Follow-Up

Our primary outcomes of interest were longitudinal BP measures, and the prevalence of HTN and hypotension, in the 8 weeks after the index date (ie, weeks 1-8 postindex) (analytical windows provided in Supplementary Figure 1). We calculated the average number of SBP/DBP measurements per day, the average SBP/DBP (in mm Hg), and the proportion of residents experiencing ≥1 hypotensive/hypertensive readings. To compare BP measures between dementia groups and pre—/post—COVID-19, we used BP measurements during the first week after the index date. We then measured BP variability from the range of SBP among those with at least 2 measures within a week. Cross sectionally, we developed a heatmap to visualize the prevalence of hypotension and HTN during follow-up, stratified by the total number of antihypertensive medication classes taken (during week 1 postindex) and baseline dementia severity.^18,19^

In a secondary analysis, we considered time to first deprescribing event among NH residents, with a focus on comparing those with dementia with those without dementia. Deprescribing was defined as any reduction in the number of antihypertensives or a reduction in an antihypertensive’s dose maintained for at least 7 consecutive days (Supplementary Table 2). In analyzing deprescribing in antihypertensive medications, we followed residents from the index date until the first of the following: (1) a deprescribing event, (2) death (measured by discharges due to death), (3) discharge from the NH (recorded discharge or >10 consecutive days without evidence of residence in the NH), (4) end of the 90-day assessment window, or (5) end of the study data (March 7, 2022).

### Covariates

Dementia was measured based on the MDS Cognitive Function Scale (CFS) and MDS comorbidity indicators for ADRD (I4200 or I4800).^20^ The CFS integrates self-reported Brief Interview for Mental Status responses with staff-reported Cognitive Performance Scale ratings. The CFS creates a hierarchical 4-level scale from 1 (no impairment) to 4 (severely impaired) using either the Brief Interview for Mental Status or Cognitive Performance Scale score. We classified residents with dementia as those with ADRD indicators checked on their baseline MDS assessment or who had a baseline CFS measurement of 3 or 4. All others were classified as not having dementia.

To explore temporal patterns and impacts on our outcomes associated with the onset of the COVID-19 pandemic, we subcategorized the cohorts into pre— and post—COVID-19 periods. The pre—COVID-19 cohort includes NH admission dates occurring between February 1, 2018, and October 3, 2020. The post—COVID-19 cohort included admissions from November 3, 2020, onward, according to the World Health Organization declaration of the outbreak as a global pandemic.^21^ We used the baseline MDS assessment to measure other covariates including age categories (<65, 65-74, 75-84, and ≥85 years), sex, race/ethnicity categories (White, Black, Hispanic, and other race or ethnicity), and comorbidities (Supplementary Table 3).

### Statistical Analysis

To compare baseline demographic and clinical characteristics between residents with and without dementia, we used absolute standardized mean differences (aSMDs), with an aSMD >0.1 (10%) indicating a meaningful imbalance between groups.^22^ For the primary analysis, aSMDs were calculated between COVID-19 periods, stratified by dementia status. The χ^2^ statistics were used to assess the statistical significance of the impact of level of dementia severity (CFS score) on hypotension and HTN prevalences.

We used Kaplan-Meier estimators to describe the unadjusted time to the first deprescribing event by dementia.^23^ Cause-specific Cox proportional hazards models were used to compare rates of deprescribing between dementia groups, treating discharge as a competing risk. Cause-specific hazard ratios (HRs) with 95% CIs were estimated and interpreted as the relative hazard of deprescribing at any given time during follow-up, among individuals still at risk.^24-26^ Four models were estimated: an unadjusted model, a demographic-adjusted model, a comorbidity-adjusted model, and a fully adjusted model to assess the association of dementia on the likelihood of deprescribing.

We conducted an additional analysis stratified by COVID-19 period to examine whether the association between dementia and deprescribing differed before and after the onset of the pandemic. All statistical analyses were conducted using SAS version 9.4. Statistical significance was set at a 2-sided alpha value of <0.05.^27^ We used ggplot and tidyr packages in R Studio to create the heatmap graphs.^28-34^

## Results

### Study Population and Baseline Characteristics

The study included 141,630 eligible NH residents (exclusion flow provided in Supplementary Figure 2), with 27.9% (n = 39,522) having evidence of dementia. Table 1 summarizes baseline demographics and clinical characteristics overall and by dementia status. Residents with dementia were on average older than those without dementia (mean age, 79.8 vs 73.7 years; aSMD, 0.54). The sex distribution was similar across groups (44% male). The median length of stay in the NH was 36 days (interquartile range, 21.0-190.0 days).

Baseline SBP/DBP values and BP monitoring frequency were similar between dementia groups, at approximately 129/71 mm Hg and 2 measures per day, respectively. Most of the cohort (58.6%) took only 1 antihypertensive medication, whereas 6.4% received ≥3 classes. Beta-blockers were most common (69.0%), followed by angiotensin-converting enzyme inhibitors (33.7%), and angiotensin-receptor blockers (21.4%). Table 2 compares BP measures during the first week after the index date, stratified by dementia status and COVID-19 periods. The average number of BP measurements per day was similar by dementia status, but for both groups increased post—COVID-19 (1.6 to 1.9 in the dementia group [aSMD, 0.24]; 1.6 to 1.9 in the no dementia group [aSMD, 0.22]). The ranges of SBP and DBP each week were large overall yet increased minimally from pre— to post—COVID-19. Despite high BP variability, average SBP/DBP remained stable across periods. There were no significant differences in hypotension or HTN prevalence by COVID-19 period for both dementia groups.

### Longitudinal Trends in BP Measurements and Hypotension and HTN

Trends in BP variability, hypotension, and HTN prevalence across the first 8 weeks after the index by dementia status and the COVID-19 period are presented in Supplementary Figure 3 and Supplementary Table 4. Both SBP and DBP variability and the prevalence of hypotensive and hypertensive events decreased throughout the 8-week follow-up period. Most differences in longitudinal measures across dementia groups and by period were relatively small. Overall, 14.2% of residents with dementia experienced at least 1 hypotensive event, with 20.9% of these having ≥3 instances of hypotension during the follow-up.

### Intersection of Number of Antihypertensive Medications, BP, and Dementia Severity

**Fig. 1.**
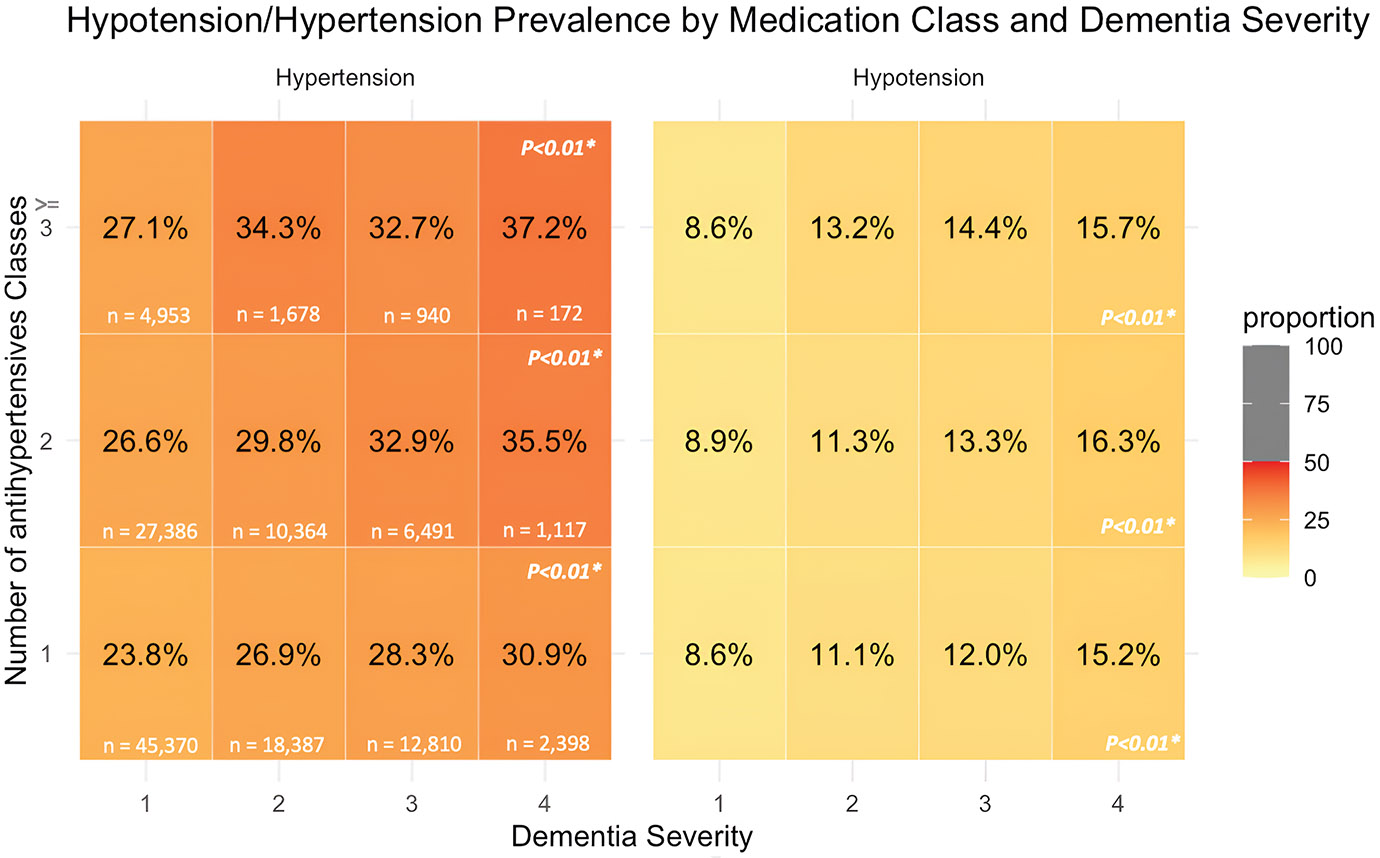
Prevalence of hypotension and HTN (%) by number of medication class administered and dementia severity. For example, among residents with a CFS score of 4 who were prescribed only 1 antihypertensive drug class (n = 2398), 15.2% experienced hypotension. (A) Dementia severity is scored on a scale from 1 to 4, with 1 indicating no or mild impairment and 4 indicating severe impairment. This categorization is based on the MDS CFS. There were 1717 missing dementia severity scores. (B) Total number of antihypertensive classes for this analysis was measured in week 1 of follow-up (in the 7 days after index). Dementia severity was obtained from baseline MDS assessment; prevalences of hypotension/HTN were calculated from weeks 2 through 8 postindex to avoid issues of temporality with measurement during the same week as the number of antihypertensive medication classes; prevalences of hypotension/HTN were calculated from week 2 to week 8 after the index. Hypertension is defined as SBP >160 mm Hg, whereas hypotension is defined as SBP <90 mm Hg for this study. (C) Sample sizes for each dementia severity and number of antihypertensive classes taken are shown in each cell. (D) The χ^2^ test *P* values were calculated for the association between the total number of medication classes administered and hypotension/HTN prevalence, controlling for each dementia severity. *P <* .05 indicates statistical significance.

Figure 1 shows the prevalence of hypotension and HTN by the number of antihypertensive medication classes taken and dementia severity (CFS score). Among those with the same number of antihypertensive drug classes taken, the proportion of residents experiencing ≥1 hypotensive/hypertensive readings both increased as dementia severity increased. For instance, among those taking ≥3 medications, hypotension prevalence rose from 8.6% in the no dementia group to 15.7% in the severe dementia group. The prevalence of HTN also increased moderately by CFS score (eg, among residents taking ≥3 medications, HTN prevalence was 27.1% in those with CFS of 1 vs 37.2% in those with CFS of 4).

### Deprescribing Rates by Dementia Status

**Fig. 2.**
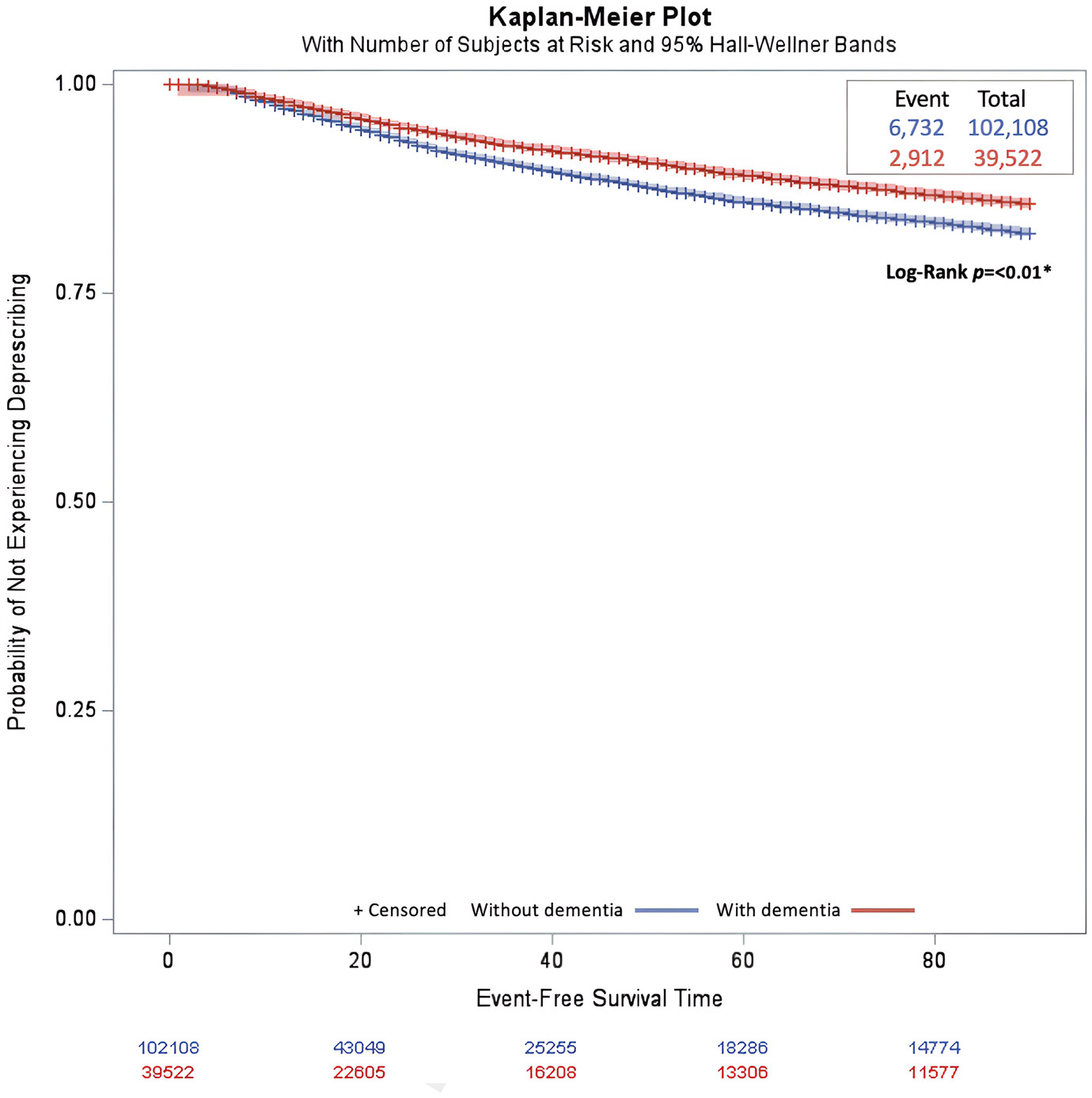
Kaplan-Meier curve for time to first deprescribing event by dementia status. “Total” indicates the number of residents at baseline; “event” indicates the number who experienced deprescribing. Numbers at risk are shown below the x-axis; + symbols indicate censoring.

A total of 9641 NH residents (6.8%) experienced antihypertensive deprescribing during an average of 31.9 days of follow-up (dementia: 41.0 days, no dementia: 27.9 days). After 90 days of follow-up, 14.4% of residents with dementia and 17.9% of those without dementia experienced deprescribing per the Kaplan-Meier estimator (Figure 2). The corresponding deprescribing rates were 1.8 and 2.4 events per 1000 person-days, respectively. Most residents were censored due to discharge from the NH (71.2%), followed by the end of the 90-day follow-up (16.8%) (Supplementary Table 5). In the unadjusted model, the HR for deprescribing for those with vs without dementia was 0.77 (95% CI, 0.74-0.81). This association remained stable after adjusting for demographics, comorbidities, and total number of antihypertensive medication classes (HR, 0.83; 95% CI, 0.79-0.86) (Table 3). In the analysis stratified by COVID-19 period, the association between dementia and the outcome remained consistent (Supplementary Table 6).

## Discussion

We conducted a longitudinal cohort study of 141,630 newly admitted NH residents treated for HTN and found that BP monitoring increased post-COVID-19. Average BP values and BP variability were similar pre—/post—COVID-19. Notably, residents with dementia—particularly those with severe dementia—were more likely to experience hypotension. Additionally, deprescribing rates were 17% lower among residents with dementia compared with those without dementia.

Our results complement findings from other health systems’ NH populations, such as a VA-based study by Odden et al,^4^ which reported high rates of HTN treatment and control at admission. Our study adds a longitudinal perspective by capturing BP monitoring patterns before and after the onset of the COVID-19 pandemic in a generalizable population of US NH residents. Specifically, we observed a slight increase in BP monitoring postpandemic among both residents with and without dementia. This uptick may reflect the implementation of enhanced monitoring protocols after the federal Centers for Disease Control and Prevention’s guidance issued on March 13, 2020, and state-level guidance recommending increased monitoring of NH residents’ vitals and symptoms to detect and manage potential COVID-19 cases.^35,36^ Characteristics of residents sent to NHs may also have changed after the COVID-19 pandemic onset, where NH admission may have been reserved for residents who had a worse health status given the increased risk of contracting SARS-CoV-2 in NHs.^37,38^ NH residents after March 11, 2020, may therefore have required more BP monitoring due to worse individual health status, in addition to the increased monitoring protocols. Further research is needed to clarify whether closer monitoring of NH residents with dementia could improve clinical outcomes because current BP monitoring protocols typically apply the same frequency to all residents regardless of cognitive status.

Unlike previous studies that found worsening cognitive impairment associated with less intensive HTN treatment,^5^ we found that residents with dementia were likely overtreated. For example, those with severe cognitive impairment experienced a higher prevalence of hypotension, and <7% of residents with dementia experienced deprescribing. These differences may reflect our inclusion of shortstay residents, different outcome definitions, and focus on the COVID-19 pandemic period, in contrast with prior studies of long-stay residents using prepandemic data.^5^ Evidence from long-term care studies suggests that more intensive antihypertensive treatment offers limited functional benefits and has been associated with modest rises in hospitalization rates and cardiovascular hospitalizations, further highlighting concerns about overtreatment.^5^ Future research should focus on understanding the long-term outcomes of antihypertensive overtreatment in NH residents with dementia and aim to establish clearer guidelines for this population.

Our study suggests that NH prescribers may be cautious when deprescribing antihypertensives for residents with dementia. The lower deprescribing rates, 1.8 and 2.4 per 1000 person-days for residents with and without dementia, respectively, observed in our study may reflect differences in clinical setting and population because our cohort included newly admitted, post-acute NH residents, whereas prior VA-based studies included long-stay or end-of-life residents for whom deprescribing is more clinically appropriate and commonly implemented.^39,40^ Although the American Geriatrics Society’s Choosing Wisely recommendations advocate for reducing medications in patients with advanced dementia or limited life expectancy, implementation in real-world practice remains inconsistent. Qualitative studies highlight enablers of deprescribing, such as multidisciplinary collaboration and improving quality of life, but also barriers such as poor communication between staff, residents, and families, and limited deprescribing tools.^41,42^ MedSafer,^43^ a deprescribing tool that integrates with PointClickCare, which applies an evidence-based set of criteria to address polypharmacy, has been shown to safely promote deprescribing in long-term care homes; however, another study found no significant impact on adverse drug events.^44,45^ Future research should assess the safety and effectiveness of less intensive HTN treatment in NH residents, especially those with dementia. Future deprescribing efforts should also consider residents’ cognitive and functional status alongside clinical events, such as falls or renal impairment, to better balance the risks and benefits of continued antihypertensive use.^40^ Addressing key elements like communication, coordination issues, and personalized reports of deprescribing development will also be essential to optimize care. Potential policy levers could include integrating deprescribing metrics into NH quality measures or requiring pharmacist-led medication reviews within 14 days of admission for residents with low SBP or symptoms like dizziness or syncope, to systematically identify candidates for antihypertensive deprescribing.

### Strengths and Limitations

Our study has several strengths. Using data from approximately 11% of all US NHs, our study provides a nationally relevant perspective on longitudinal HTN management in NH residents with and without dementia with high generalizability. We also used a rigorous definition of deprescribing that incorporated and required sustained changes for at least a week to exclude transient medication fluctuations and capture meaningful trends. However, several limitations must be considered when interpreting our results. First, death records were not obtained or linked to Medicare data, which could lead to misclassification and underestimation of mortality outcomes. Second, our study does not distinguish between prevalent and new users of antihypertensives, but deprescribing rates may vary based on treatment history. Third, many residents were discharged during follow-up, so our analysis captures only early deprescribing. Finally, we were unable to account for facility-level BP monitoring protocols, which may influence both the frequency of BP assessments and the likelihood of detecting elevated readings that trigger treatment changes.

## Conclusions and Implications

BP monitoring increased for NH residents after the onset of the COVID-19 pandemic. Despite more frequent monitoring, there was a high prevalence of both HTN and hypotension, particularly among residents with dementia. These results underscore the need for tailored deprescribing strategies and cautious BP management to avoid overtreatment and minimize harm in cognitively impaired NH populations. Future research should focus on optimizing antihypertensive treatment and deprescribing practices to balance BP control with quality of life in this vulnerable group.

## Supplementary Material

Supplement

Supplementary data related to this article can be found online at https://doi.org/10.1016/j.jamda.2025.105883.

## Figures and Tables

**Table 1:** Characteristics of NH Residents admitted January 2018 to July 2022 Receiving Antihypertensive Medications, Stratified by Dementia Status

Characteristics	All (N = 141,630)	Dementia		aSDM*
		No (n = 102,108; 72.1%)	Yes (n = 39,522; 27.9%)	
Age, y	75.4 ± 12.0	73.7 ± 12.1	79.8 ± 10.6	**0.54** *
Age categories				**0.52** *
<65 y	17.9	21.5	8.6	
65-74 y	26.2	29.2	18.5	
75-84 y	31.2	29.3	35.9	
≥85 y	24.7	20.0	37.0	
Sex, male	43.3	43.6	42.6	0.02
Race/ethnicity				0.08
White	73.4	74.3	70.8	
Black	15.6	14.8	17.7	
Hispanic	1.9	1.7	2.4	
Other	9.1	9.2	9.1	
NH length of stays, d	195.9 (21.0, 36.0, 190.0)	121.1 (20.0, 30.0, 61.0)	218.3 (24.0, 44.0, 231.0)	**0.30** *
SBP				
Averages, mm Hg	129.3 ± 11.4	129.3 ± 11.5	129.5 ± 11.1	0.03
Readings, number per day	2.0 ± 1.2	2.0 ± 1.2	1.9 ± 1.2	0.07
DBP				
Averages, mm Hg	71.0 ± 6.4	70.9 ± 6.5	71.2 ± 6.2	0.05
Readings, number per day	2.0 ± 1.2	2.0 ± 1.2	1.9 ± 1.2	0.07
Antihypertensive drug classes used				
Angiotensin-converting enzyme inhibitors	33.7	33.3	34.4	0.02
Angiotensin-receptor blockers	21.4	21.8	20.4	0.03
Calcium channel blockers	10.9	11.1	10.3	0.03
Thiazide diuretics	13.4	14.3	10.8	**0.11** *
Beta-blockers	69.0	69.5	67.7	0.04
Number of antihypertensive drugs class, averages				**0.12** *
1	58.6	57.3	62.0	
2	35.0	35.8	32.7	
≥3	6.4	6.9	5.3	
Baseline comorbidities				
Diabetes	45.3	47.2	40.3	**0.14** *
Stroke	13.4	11.4	18.7	**0.21** *
Heart failure	28.4	29.8	24.8	**0.11** *
Renal disease	31.8	32.5	30.0	0.05
MI	27.2	27.2	27.1	<0.01

MI, myocardial infarction.

Values are mean ± SD, %, mean (quartile 1, median, quartile 3), or as otherwise indicated.

* An aSMD of 0.1 (10%) indicates a meaningful imbalance.

**Table 2:** BP Control During the First Week After the Index Date by Dementia Status and COVID-19 Period

Admission	Dementia	Number of Residents	Range of SBP*^,†^	Range of DBP*^,†^	Average SBP/DBP Measures per Day	Average SBP/DBP (mm Hg)	Hypotension/ Hypertension^‡^ (%)
Pre—COVID-19	No	59,607	37.4 (24, 36, 49)	23.6 (16,23,30)	1.6 (1.2)	128.9 (13.5)/70.5 (7.6)	4.5/22.1
	Yes	22,691	38.3 (24, 37, 51)	24.0 (16,24,31)	1.6 (1.2)	128.9 (13.1)/70.6 (7.3)	4.8/23.0
Post—COVID-19	No	42,501	38.8 (26, 38, 51)	25.0 (18,25,32)	1.9 (1.3)	128.4 (12.5)/70.7 (7.3)	4.7/21.9
	Yes	16,831	40.0 (27, 40, 53)	25.8 (18,26,33)	1.9 (1.3)	128.0 (12.2)/70.8 (6.9)	5.2/22.9
aSMD^§^ by COVID-19 period		ADRD = 0	0.09	**0.13** *	**0.22** *	0.04/0.03	0.01/<0.01
		ADRD = 1	**0.12** *	**0.18** *	**0.24** *	0.07/0.02	<0.02/<0.01

* The Number of residents for the range of SBP/DBP includes only residents with ≥2 total SBP/DBP readings during the first week after the index. Specifically, the numbers are 17,832 pre—COVID-19 with dementia, 48,199 pre—COVID-19 without dementia, 13,797 post—COVID-19 with dementia, and 34,849 post—COVID-19 without dementia.

† Range of SBP/DBP indicates mean (25th percentile, median, 75th percentile). Note that SBP and DBP were capped at their 99th percentiles, 180 and 98, respectively.

‡ Hypertension was defined as having an SBP >160 mm Hg, and hypotension was defined as an SBP <90 mm Hg.

§ An aSMD of 0.1 (10%) indicates a meaningful imbalance.

**Table 3:** HRs for the First Deprescribing Event in Cause-Specific Cox Proportional Hazards Models

Variable	HR (95% CI)			
	Model 1 Unadjusted*	Model 2	Model 3 − Model 2 + Comorbidities^‡^	Model 4 − Model 3 +
	
		Demographics^†^		Number of Medication Class^§^
Dementia	0.77 (0.74, 0.81)	0.75 (0.72-0.78)	0.78 (0.75-0.82)	0.83 (0.79-0.86)
Age, y				
<65 (reference)	—	—	—	—
65-74	—	1.16 (1.09-1.24)	1.12 (1.05-1.19)	1.11 (1.04-1.18)
75-84	—	1.29 (1.21-1.37)	1.23 (1.16-1.31)	1.20 (1.13-1.27)
≥85	—	1.28 (1.20-1.36)	1.21 (1.14-1.30)	1.21 (1.14-1.30)
Sex	—	0.90 (0.86-0.93)	0.92 (0.89-0.96)	0.90 (0.86-0.94)
Race/ethnicity				
White (reference)	—	—	—	—
Black	—	0.83 (0.78-0.88)	0.82 (0.77-0.86)	0.79 (0.75-0.84)
Hispanic	—	0.89 (0.77-1.03)	0.90 (0.77-1.04)	0.89 (0.76-1.03)
Others	—	1.05 (0.97-1.12)	1.06 (0.98-1.13)	1.06 (0.99-1.14)
Diabetes	—	—	1.05 (1.01-1.09)	0.97 (0.93-1.01)
Stroke	—	—	1.01 (0.96-1.07)	0.96 (0.90-1.01)
Heart failure	—	—	1.43 (1.37-1.49)	1.37 (1.31-1.43)
Renal failure	—	—	1.21 (1.16-1.26)	1.30 (1.24-1.35)
MI	—	—	1.13 (1.08-1.18)	1.09 (1.04-1.14)
Number of medications				
1 (reference)	—	—	—	—
2	—	—	—	2.82 (2.69-2.94)
3	—		—	4.41 (4.12-4.71)
4	—		—	6.69 (5.46-8.06)
5	—		—	4.25 (0.24-18.71)

MI, myocardial infarction.

∥ Ten individuals were excluded from models 3 and 4 due to missing comorbidity data: diabetes (n = 1), heart failure (n = 5), renal failure (n = 3), and MI (n = 1).

* Model 1 was unadjusted (N = 141,630).

† Model 2 was adjusted for demographics including age categories (reference: <65 y) (ie, <65, 65-74, 75-84, ≥85 y), sex (reference: male), and race/ethnicity categories (reference: White) (ie, White, Black, Hispanic, others) (N = 141,630).

‡ Model 3 was adjusted, including the variables from model 2 and comorbidities including diabetes (reference: no), stroke (reference: no), heart failure (reference: no), renal failure (reference: no), and MI (reference: no) (n = 141,620).

§ Model 4 was fully adjusted, including the variables from model 3 and total number of antihypertensives classes taken (reference: 1) (n = 141,620).
