# Supplementary material for: Hypertension Treatment, Blood Pressure, and Deprescribing Among US Nursing Home Residents With and Without Dementia Before and After the COVID-19 Pandemic
Source: J Am Med Dir Assoc. Author manuscript; Available in PMC 2025 Nov 4. (PMC12581180; doi:10.1016/j.jamda.2025.105883)
Supplement: Supplement [file NIHMS2115314-supplement-Supplement.docx]

**SUPPLEMENTARY MATERIAL**

***Hypertension Treatment, Blood Pressure, and Deprescribing Among U.S. Nursing Home Residents With and Without Dementia Before and After the COVID-19 Pandemic***

Supplementary Table 1. Antihypertensive Medication Classes

| **First-line antihypertensive medication class** | **Drug Names Included** |
| --- | --- |
| Angiotensin-Converting Enzyme Inhibitors | Lisinopril, Zestril, Qbrelis, Prinzide, Prinvil, Zestoretic, Tarka, Enalapril, Vasotec, Epaned, Teczem, Lexxel, Vaseretic, Benazepril, Lotensin, Lotrel, Amlobenz, Lotensin Hct, Fosinopril, Monopril, Captopril, Capoten, Captoril, Capozide, Moexipril, Univasc, Perindopril, Aceon, Coversyl, Coversum, Prestalia, Quinapril, Accupril, Accuretic, Quinaretic, Trandolapril, Mavik, Ramipril, Altace. |
| Angiotensin-Receptor Blockers | Valsartan, Losartan, Cozaar, Hyzaar, Candesartan, Atacand, Diovan, Prexxartan, Valturna, Exforge, Byvalson, Entresto, Eprosartan, Teveten, Azilsartan, Edarbi, Olmesartan, Benicar, Tribenzor, Azor, Telmisartan, Micardis, Twynsta, Irbesartan, Edarbyclor, Avapro. |
| Calcium Channel Blockers | Diltiazem, Verapamil, Cardizem, Cartia, Dilt-xr, Felodipine, Cabren, Cardioplen, Felendil, Felogen, Felotens, Keloc, Neofel, Plendil, Renedil, Nicardipine, Cardene, Israpidine, Dynacirc, Prescal, Isoptin, Verelan, Calan, Tarka, Nifedipine, Procardia, Adalat, Nifedical, Nisoldipine, Sular, Teczem, Lexxel, Lotrel, Amlobenz, Exforge, Azor, Twynsta, Amturnide, Tekturna. |
| Thiazide Diuretics | Hydrochlorothiazide, Thiazide, Hct, Chlorothiazide, Diuril, Aldoclor, Diupres, Chlorthalidone, Hygroton, Thalitone, Chlorthalid, Edarbyclor, Clorpres, Regroton, Microzide, Hydrodiuril, Esidrix, Oretic, Amturnide, Tekturna, Moduretic, Oreticyl, Esimil, Aldoril, Aldactazide, Dyazide, Maxzide, Indapamide, Lozol, Methyclothiazide, Aquatensen, Enduro, Metolazone, Zaroxolyn, Mykrox, Prinzide, Zestoretic, Vaseretic, Capozide, Accuretic, Quinaretic, Hyzaar, Tribenzor, Avalide, Dutoprol, Minizide, Diuretic, Ap-es, Serpex, Hydrap-es, Tenoretic, Corzide, Serathide. |
| Beta Blockers | Metoprolol, Labetalol, Acebutolol, Sectral, Atenolol, Tenormin, Tenoretic, Betaxolol, Bisoprolol, Monocor, Zebeta, Ziac, Carvedilol, Coreg, Trandate, Normodyne, Lopressor, Toprol, Dutoprol, Nadolol, Corgard, Corzide, Nebivolol, Bystolic, Pindolol, Visken, Propanolol, Inderal, Innopran, Byvalson, Hemangeol. |

Supplementary Table 2.1. Deprescribing Text Extraction Examples - **Original**

| patient_ID | drug_name | schedule_directions | drug_strength | dose |
| --- | --- | --- | --- | --- |
| A | Hydralazine HCl | Give **2** tablet by mouth **two times** a day for Heart Health related to ESSENTIAL (PRIMARY) HYPERTENSION | 25 | 2 |
| B | Metoprolol Tartrate | Give **100 mg** by mouth **two times** a day for Hypertension | 50 | 100 |
| C | Metoprolol Tartrate | Give **1** tablet by mouth **every morning** and **at bedtime** for HTN | 25 | 1 |

**Logics**:

### **1. corrected_dose**

- Extracts the dose information based on the text following the word "Give".
- If units like mg, mmg, or mcg are found in the text, corrected_dose is assigned the value of dose.
- If no such units are found, it falls back to drug_strength (if available) or defaults to dose.

### **2. corrected_freq1**

- Represents a modifier based on whether the dose units (mg, mcg, etc.) are present.
- Defaults to 1 when units are found (dose is treated as per schedule) or takes the value of dose (number of tables/capsules) otherwise.

### **3. corrected_freq2**

- Looks for keywords like two, three, etc., followed by time or times in the directions.
- Assigns a numeric equivalent (e.g., two → 2).
- Defaults to 1 if no such pattern is found (indicating a single instance).

**4. corrected_freq3**

- Captures additional frequency details based on specific time-related phrases:
  - Adds 1 for "morning".
  - Adds 1 for "bedtime".
  - Adds 1 for "evening".
  - Adds 1 for "afternoon".
- Defaults to 1 if no matching phrases are found.

**4. final_dose =** corrected_dose ✕ corrected_freq1 ✕ corrected_freq2 ✕ corrected_freq3

Supplementary Table 2.2. Deprescribing Text Extraction Examples

| resident_ID | corrected_dose | corrected_freq1 | corrected_freq2 | corrected_freq3 | final_dose |
| --- | --- | --- | --- | --- | --- |
| A | 25 | 2 | 2 | 1 | 100 |
| B | 100 | 1 | 2 | 1 | 200 |
| C | 25 | 1 | 1 | 2 | 25 |

Supplementary Table 3. Covariate Domains, Data Sources, and Example Measures

| **Data Domain** | **Data Source** | **Example Measures** |
| --- | --- | --- |
| Demographics | NH EHR-MDS | Age (A0900), sex (A0800), race/ethnicity (A1000) |
| Comorbidities and Multimorbidity | NH EHR-MDS | Diabetes (I2900), Stroke (I4500), Heart failure (I0600), Renal disease (I1500), MI (I0400) |
| Medication Use, Polypharmacy | NH EHR - eMAR | Current antihypertensive medication use (classes and count of medications) |
| Vital Signs | NH EHR | SBP, DBP |

Supplementary Figure 1. Analytic Windows


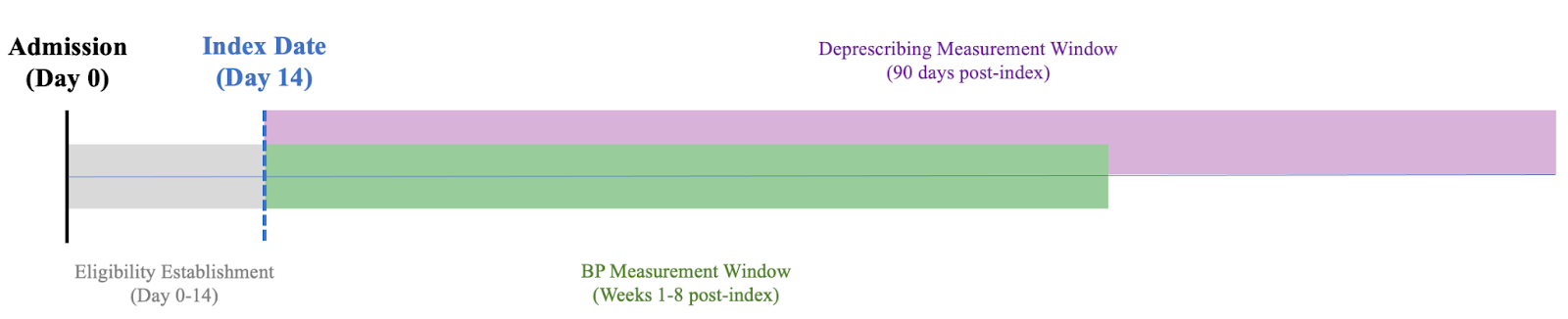


**Abbreviations:** BP: Blood Pressure.

**Note:** The findings should not be generalized to all NH residents, particularly those who experienced discharge, death, or hospice enrollment within 14 days of admission, as they were excluded from the analysis.

Supplementary Figure 2. Study Exclusion Flow


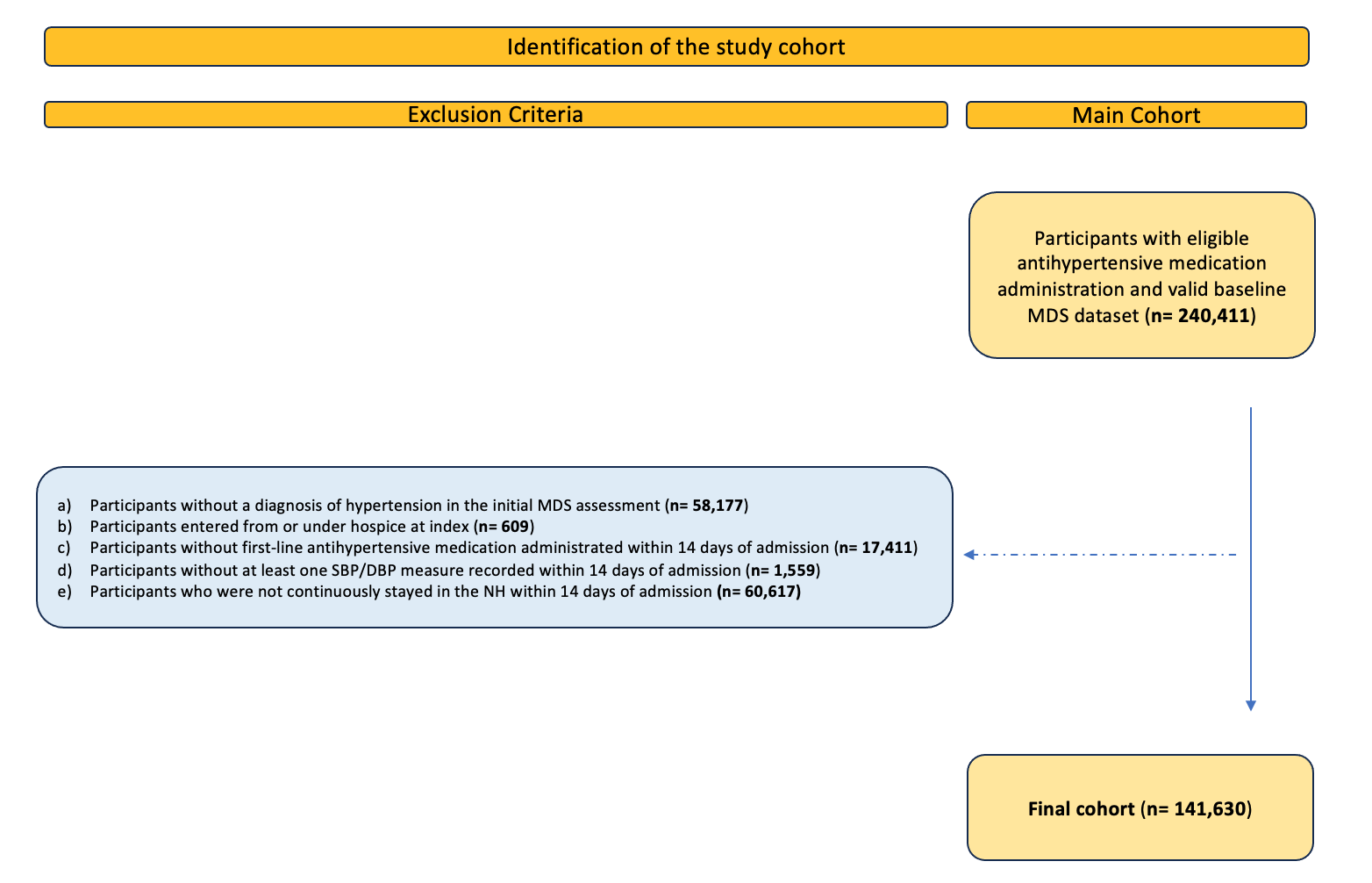


Supplementary Table 4. Statistics of SBP range, Hypotension Prevalence, and Hypertension Prevalence by Dementia Status and COVID-19 Period

**SBP Range (mmHg, Mean ± SD) During 8-Week Follow-Up in the Pre-COVID Period By Dementia Status**

|  | **No (N= 59,607)** | **Yes (N= 22,691)** |
| --- | --- | --- |
| Week 1 | 37.5 (19.3) | 38.4 (20.2) |
| Week 2 | 37.4 (19.7) | 37.6 (20.3) |
| Week 3 | 37.2 (19.6) | 37.2 (20.3) |
| Week 4 | 37.1 (19.8) | 36.4 (19.8) |
| Week 5 | 37.0 (19.6) | 36.4 (19.8) |
| Week 6 | 37.2 (19.5) | 36.1 (20.0) |
| Week 7 | 36.8 (19.8) | 36.6 (20.1) |
| Week 8 | 37.1 (20.0) | 35.7 (20.4) |

**SBP Range (mmHg, Mean ± SD) During 8-Week Follow-Up in the Post-COVID Period By Dementia Status**

|  | **No (N= 42,501)** | **Yes (N= 16,831)** |
| --- | --- | --- |
| Week 1 | 38.9 (19.0) | 40.1 (19.4) |
| Week 2 | 38.5 (19.1) | 39.3 (19.5) |
| Week 3 | 38.5 (19.3) | 39.0 (19.9) |
| Week 4 | 38.5 (19.3) | 38.9 (19.8) |
| Week 5 | 38.4 (19.0) | 38.8 (19.9) |
| Week 6 | 38.6 (19.1) | 39.2 (20.2) |
| Week 7 | 38.2 (19.1) | 38.8 (19.8) |
| Week 8 | 38.5 (19.6) | 38.3 (20.0) |

**Hypo-/Hypertension Prevalences During 8-Week Follow-Up in the Pre-COVID Period By Dementia Status**

|  | Hypotension | | Hypertension | |
| --- | --- | --- | --- | --- |
|  | **No (N= 59,607)** | **Yes (N= 22,691)** | **No (N= 59,607)** | **Yes (N= 22,691)** |
| Week 1 | 4.9 | 5.2 | 23.6 | 24.8 |
| Week 2 | 5.1 | 5.2 | 24.0 | 24.0 |
| Week 3 | 4.8 | 5.0 | 23.5 | 23.7 |
| Week 4 | 5.2 | 4.7 | 23.2 | 22.4 |
| Week 5 | 4.9 | 4.7 | 23.7 | 23.2 |
| Week 6 | 5.1 | 4.7 | 24.0 | 23.2 |
| Week 7 | 4.9 | 4.4 | 23.6 | 23.6 |
| Week 8 | 5.1 | 4.3 | 26.1 | 26.0 |

**Hypo-/Hypertension Prevalences During 8-Week Follow-Up in the Post-COVID Period By Dementia Status**

|  | Hypotension | | Hypertension | |
| --- | --- | --- | --- | --- |
|  | **No (N= 42,501)** | **Yes (N= 16,831)** | **No (N= 42,501)** | **Yes (N= 16,831)** |
| Week 1 | 5.0 | 5.5 | 23.1 | 24.2 |
| Week 2 | 5.1 | 5.4 | 23.0 | 23.7 |
| Week 3 | 4.9 | 5.5 | 23.1 | 23.4 |
| Week 4 | 5.2 | 5.6 | 23.0 | 23.2 |
| Week 5 | 5.3 | 5.5 | 22.7 | 23.3 |
| Week 6 | 5.1 | 5.2 | 23.5 | 24.0 |
| Week 7 | 4.9 | 5.5 | 22.9 | 23.2 |
| Week 8 | 5.4 | 5.5 | 25.6 | 25.8 |

Supplementary Figure 3. Trends in SBP range (mmHg), Hypotension Prevalence (%) and Hypertension Prevalence (%) by Dementia Status and COVID-19 Period

**
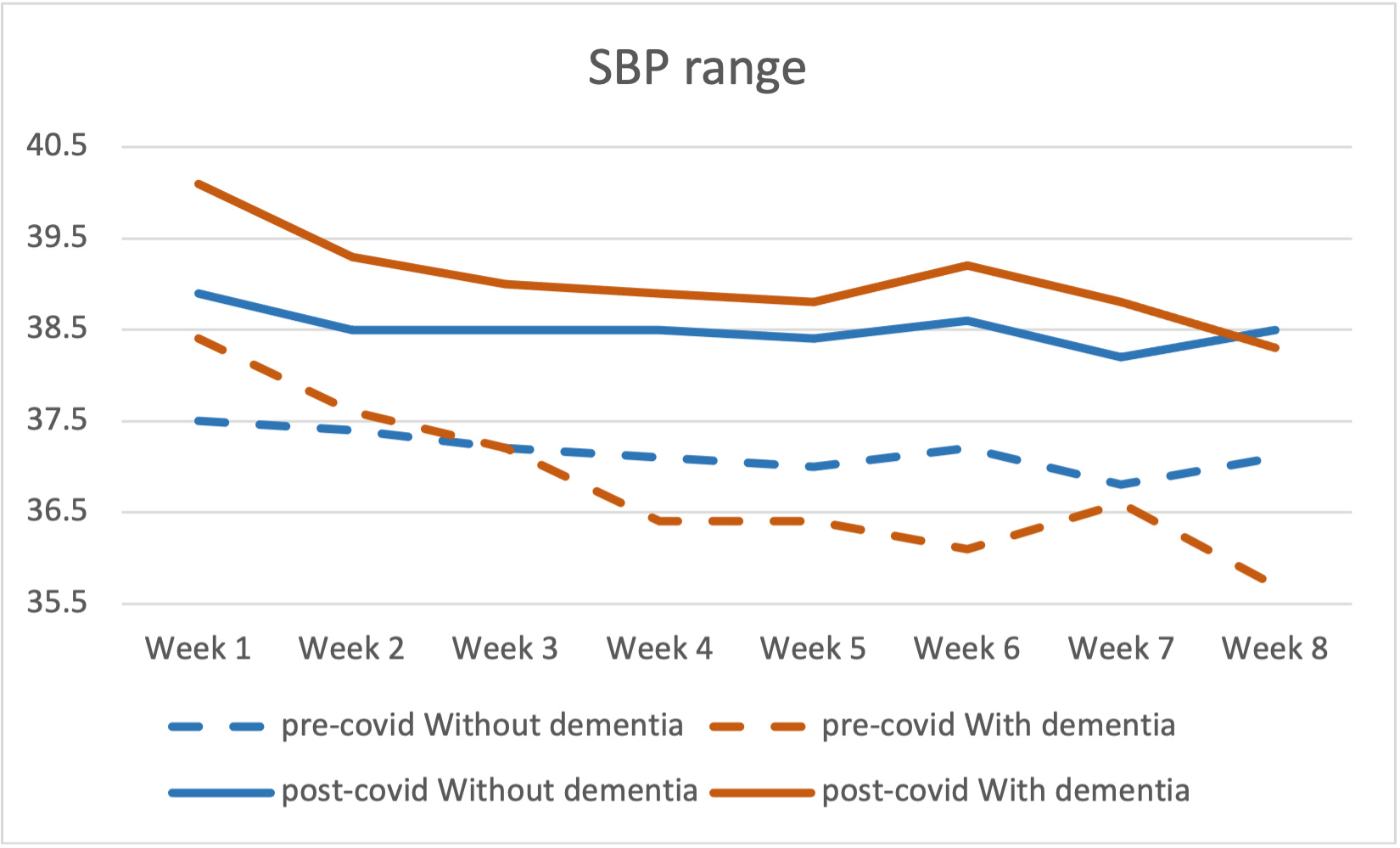
**

**Abbreviations**: SBP: Systolic Blood Pressure.

**
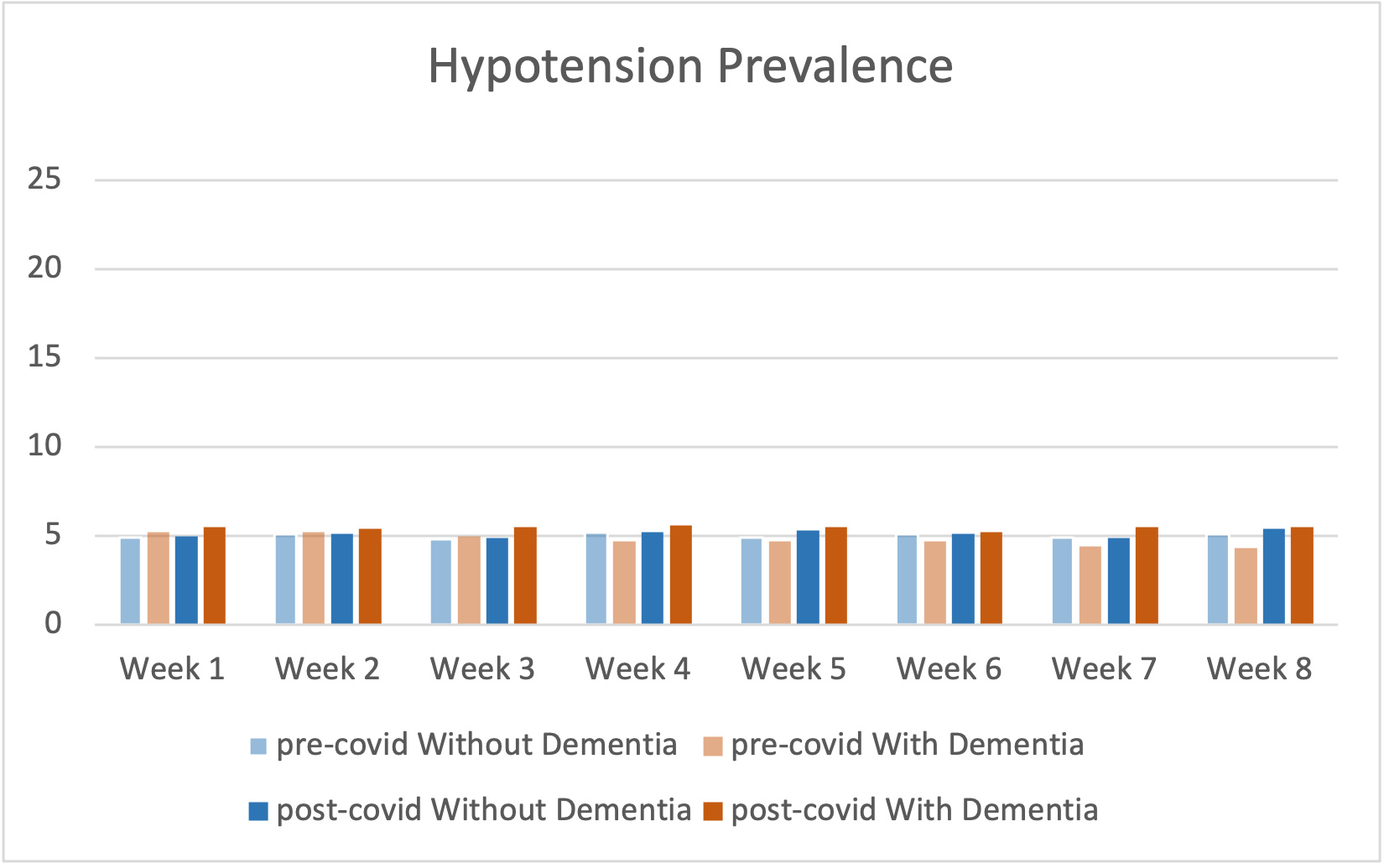
**

**
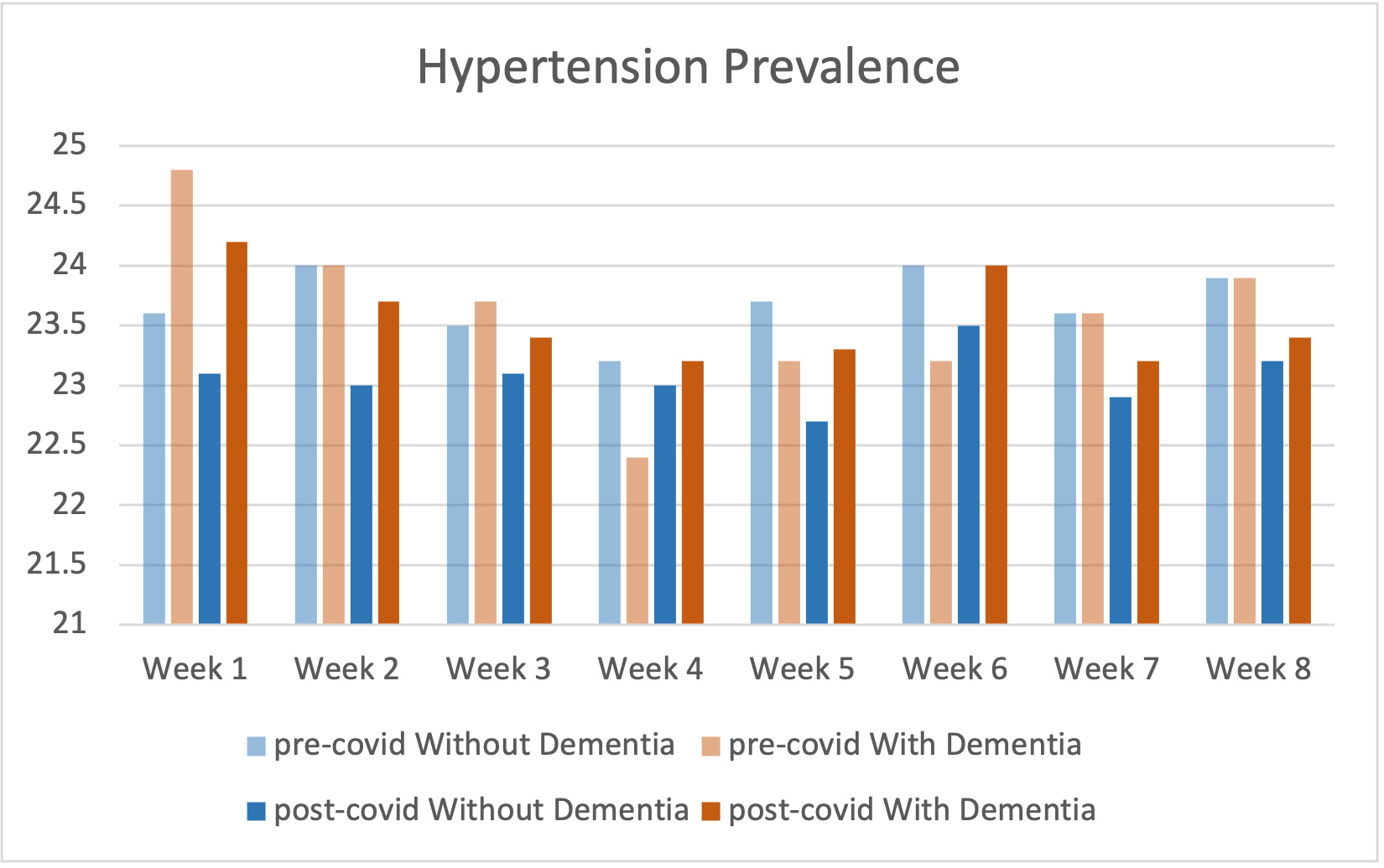
**

Supplementary Table 5. Kaplan-Meier Curve Attrition Table

| Events | **Dementia Status** | |
| --- | --- | --- |
|  | **No (N= 102,108 [72.1%])** | **Yes (N= 39,522 [27.9%])** |
| The first deprescribing | 6,730 (6.6) | 2,911 (7.4) |
| Death | 2,447 (2.4) | 2,735 (6.9) |
| Discharged from NH | 78,394 (76.8) | 22,433 (56.8) |
| End of 90-day window | 13,106 (12.8) | 10,729 (27.2) |
| End of study | 1,431 (1.4) | 714 (1.8) |

Supplementary Table 6. Additional Analysis: Stratifying Models by COVID-19 Period

|  | **HR (95% CI)** | | | | | | | |
| --- | --- | --- | --- | --- | --- | --- | --- | --- |
| **Variable** | Model 1  - Unadjusted^a^ | | Model 2  - Demographics^b^ | | Model 3 - Model 2 + Comorbidities^c^ | | Model 4 - Model 3 +  No. of meds class^d^ | |
| **COVID-Period** | **Pre-** | **Post-** | **Pre-** | **Post-** | **Pre-** | **Post-** | **Pre-** | **Post-** |
| **Dementia** | 0.76  (0.72, 0.81) | 0.79  (0.74, 0.84) | 0.74  (0.69, 0.78) | 0.77  (0.72, 0.82) | 0.77  (0.73, 0.82) | 0.80  (0.75, 0.86) | 0.81  (0.77, 0.86) | 0.84  (0.78, 0.90) |
| **Age** |  |  |  | |  | |  | |
| <65 years (ref) | — | — | — | — | — | — | — | — |
| 65-74 years | — | — | 1.13  (1.05, 1.23) | 1.20  (1.09, 1.33) | 1.09  (1.01, 1.19) | 1.16  (1.05, 1.28) | 1.08  (0.99, 1.17) | 1.15  (1.04, 1.26) |
| 75-84 years | — | — | 1.30  (1.20, 1.41) | 1.28  (1.16, 1.41) | 1.24  (1.14, 1.34) | 1.22  (1.10, 1.34) | 1.21  (1.11, 1.31) | 1.18  (1.08, 1.31) |
| >=85 years | — | — | 1.27  (1.17, 1.39) | 1.28  (1.16, 1.42) | 1.21  (1.11, 1.32) | 1.21  (1.09, 1.34) | 1.23  (1.13, 1.34) | 1.19  (1.07, 1.32) |
| **Sex** | — | — | 0.90  (0.86, 0.95) | 0.89  (0.83, 0.94) | 0.93  (0.88, 0.98) | 0.92  (0.86, 0.98) | 0.90  (0.86, 0.95) | 0.89  (0.84, 0.95) |
| **Race/Ethnicity** |  | |  | |  | |  | |
| White (ref) | — | — | — | — | — | — | — | — |
| Black | — | — | 0.86  (0.80, 0.93) | 0.78  (0.71, 0,86) | 0.85  (0.78, 0.91) | 0.77  (0.70, 0.84) | 0.83  (0.77, 0.90) | 0.73  (0.66, 0.80) |
| Hispanic | — | — | 0.98  (0.80, 1.18) | 0.79  (0.62, 1.00) | 0.98  (0.80, 1.18) | 0.80  (0.62, 1.01) | 0.99  (0.81, 1.19) | 0.77  (0.60, 0.97) |
| Others | — | — | 1.04  (0.95, 1.15) | 1.05  (0.95, 1.17) | 1.05  (0.95, 1.15) | 1.06  (0.95, 1.18) | 1.07  (0.97, 1.17) | 1.05  (0.94, 1.17) |
| **Diabetes** | — | — | — | — | 1.06  (1.00, 1.12) | 1.03  (0.96, 1.10) | 0.98  (0.93, 1.03) | 0.96  (0.90, 1.03) |
| **Stroke** | — | — | — | — | 1.01  (0.93, 1.09) | 1.03  (0.95, 1.12) | 0.96  (0.89, 1.04) | 0.97  (0.89, 1.05) |
| **Heart Failure** | — | — | — | — | 1.44  (1.37, 1.53) | 1.40  (1.31, 1.50) | 1.39  (1.31, 1.47) | 1.34  (1.25, 1.43) |
| **Renal Failure** | — | — | — | — | 1.24  (1.17, 1.32) | 1.17  (1.10, 1.25) | 1.34  (1.27, 1.42) | 1.25  (1.17, 1.33) |
| **MI** | — | — | — | — | 1.11  (1.04, 1.17) | 1.16  (1.08, 1.24) | 1.06  (1.00, 1.13) | 1.12  (1.04, 1.20) |
| **No. of meds** |  | |  | |  | |  | |
| 1 (ref) | — | — | — | — | — | — | — | — |
| 2 | — | — | — | — | — | — | 2.80  (2.64, 2.97) | 2.85  (2.66, 3.05) |
| 3 | — | — | — | — | — | — | 4.45  (4.98, 4.84) | 4.35  (3.91, 4.82) |
| 4 | — | — | — | — | — | — | 6.10  (4.62, 7.87) | 7.53  (5.58, 9.90) |
| 5 | — | — | — | — | — | — | 7.28  (0.41, 31.96) | — |

**Abbreviations**: MI: Myocardial Infarction; No.: Number.
